# Can Optimizing the Mechanical Environment Deliver a Clinically Significant Reduction in Fracture Healing Time?

**DOI:** 10.3390/biomedicines9060691

**Published:** 2021-06-18

**Authors:** Jan Barcik, Devakara R. Epari

**Affiliations:** 1 AO Research Institute Davos, Clavadelerstrasse 8, 7270 Davos, Switzerland; 2 Bulgarian Academy of Sciences, Institute of Metal Science “Acad. A. Balevski”, Shipchenski prohod 67, 1574 Sofia, Bulgaria; 3 Institute of Health and Biomedical Innovation, Queensland University of Technology, George Street 2, Brisbane, QLD 4000, Australia; d.epari@qut.edu.au

**Keywords:** fracture healing, bone repair, mechanobiology, implants, rehabilitation

## Abstract

The impact of the local mechanical environment in the fracture gap on the bone healing process has been extensively investigated. Whilst it is widely accepted that mechanical stimulation is integral to callus formation and secondary bone healing, treatment strategies that aim to harness that potential are rare. In fact, the current clinical practice with an initially partial or non-weight-bearing approach appears to contradict the findings from animal experiments that early mechanical stimulation is critical. Therefore, we posed the question as to whether optimizing the mechanical environment over the course of healing can deliver a clinically significant reduction in fracture healing time. In reviewing the evidence from pre-clinical studies that investigate the influence of mechanics on bone healing, we formulate a hypothesis for the stimulation protocol which has the potential to shorten healing time. The protocol involves confining stimulation predominantly to the proliferative phase of healing and including adequate rest periods between applications of stimulation.

## 1. Introduction

The biomechanics of fracture healing has been scientifically investigated for at least the past 70 years [1]. Multiple studies have investigated the influence of local mechanical conditions on the progression of fracture healing; however, clinical translation of this understanding is limited. Therefore, we asked whether there is still potential for biomechanics to make substantial improvements to the clinical treatment of fractures. Subsequently, we posed the question whether there is potential to significantly shorten the healing time through optimization of the mechanical environment over the course of healing?

In this article, we predominantly examine the influence of the mechanical environment on secondary healing in the diaphysis of long bones, as this represents the condition most often studied in pre-clinical models. However, limited studies indicate that metaphyseal healing is governed by similar biomechanical rules [2]. Secondary healing is characterized by the formation of an external callus that overgrows a fracture gap to stabilize it and that later remodels into bone. For ease of reference, the process may be divided into four consecutive phases: Inflammation, proliferation, consolidation and remodelling. However, in reality these phases overlap to some degree.

The trauma to the bone and formation of haematoma leads to an initial healing reaction; however, in the absence of further mechanical stimulation, healing may discontinue after some time before the fracture has healed [3]. If, however, excessive movement persists, a delayed healing [4] or non-union (hypertrophic) [5] will occur and in prolonged cases is characterised by the formation of pseudarthrosis (false joint). Therefore, it is clear that a moderate level of mechanical stimulation (interfragmentary movement) is required to induce callus formation and, ultimately, bony union. In the natural healing process, the initial level of mechanical stimulation is controlled by the extent of functional limb loading and any stabilization applied to the fracture. In order for the opposing surfaces of the fracture bone to unite with a solid bony bridge, the relative movement of fracture fragments must be brought to negligible levels. During an uneventful healing, the initial magnitude of mechanical stimulation is gradually reduced by increasing callus size and density which increases the stiffness of the healing tissues. According to Perren’s Interfragmentary Strain Theory, the formation of successively stiffer tissues in the fracture callus reduces the fracture motion and strain, permitting progressively stiffer materials to form until bone is formed, bridging the fracture [6].

Due to its clinical significance, much research has focused on understanding the influence and optimizing the mechanical conditions provided by different passive fixation devices. With passive fixation, the interfragmentary motion at the fracture gap is determined by the stiffness of the implant and the extent of limb loading. The stiffness of a passive fixator may be constant over the whole period of a study [7,8,9] (“set and forget” approach) or adjusted during the course of healing [10,11,12]. Research has also investigated more invasive manipulation of the fixation conditions through active fixators [13,14,15,16,17]. Active fixators are equipped with an actuator that mechanically stimulates the fracture gap regardless of the animal’s activity. However, even with active fixators, the mechanical environment in the fracture gap is still influenced by functional loading [17]. The research until now has addressed the flexibility of fixation [4] or magnitude of the initial interfragmentary movements [7,18], the size of the fracture gap [7], the direction of movements [15,19,20,21], the strain rate [14] and the number [15,16] and distribution of stimulation cycles [14,17] as mechanical parameters that may affect healing.

Over recent decades of pre-clinical experiments, we have learned that high initial amplitude of stimulation leads to more pronounced callus formation [7,22] but not necessarily shorter healing time [22]. In addition, compressive stimulation is superior to tensile stimulation [15,20], although there is no consensus about the effect of shear stimulation [19,21]. Finally, a higher number of stimulation cycles induces larger callus formation [15]; however, this trend is not necessarily reflected in increasing fracture stiffness [15,16] and too frequent stimulation inhibits callus formation [16].

## 2. The Course of Fracture Healing

Post-mortem mechanical testing at the end point of a study is the gold standard for assessing healing in preclinical models. However, in order to understand the influence of mechanics on healing progression, it is necessary to investigate the evolution of the mechanical competence of the healing tissue during the course of fracture healing.

**Figure 1:**
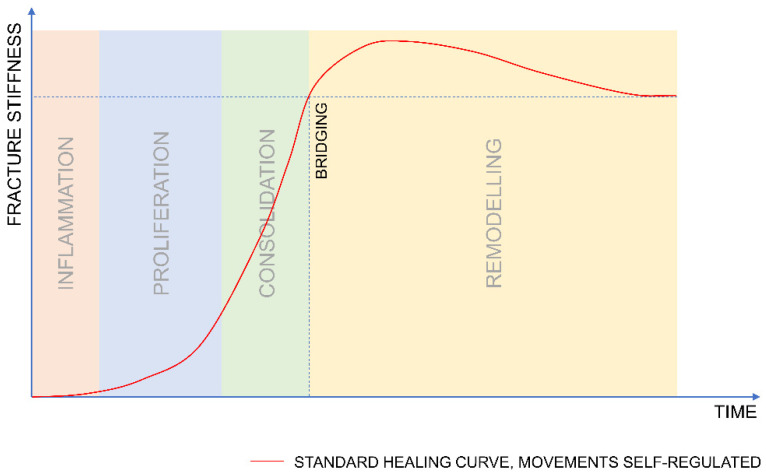
Pictorial representation of standard healing curve. After the initially slow increase of stiffness, the curve deflects and the rate at which stiffness increases accelerates. Bridging occurs when the fracture stiffness reaches the level of stiffness that allows for unrestricted functional loading of the bone (dashed line).

During the healing process, the fracture stiffness typically increases according to an S-shaped or sigmoidal curve [23,24]. Initially, fracture stiffness increases with a low rate; subsequently, the healing curve deflects and the rate at which stiffness increases accelerates. The timing of this deflection point may be correlated with the transition between the proliferation and consolidation phases. During these phases, the growth of callus and increase in stiffness self-regulates the amplitude of the interfragmentary motion (assuming unrestricted weight bearing). When bridging occurs, the fracture stiffness overshoots the value of the stiffness of an intact bone. Subsequently, the fracture stiffness reduces to pre-injury levels as the fracture callus resorbs and remodels into mature bone (Figure 1).

### 2.1. When Should Mechanical Stimulation Be Applied?

While the initial amplitude of mechanical stimulation has been identified as a crucial factor for inducing callus formation [7,9], several recent studies have questioned whether stimulation should be applied at all stages of healing [12,17,25,26]. Therefore, we have summarized what we know about the impact of stimulation during different healing phases. However, it is technically challenging to precisely identify the effect of mechanical stimulation during any particular phase of healing because it requires the continuous monitoring of healing progression [27] or multiple timepoints of sacrifice [4] which have been infrequently implemented in the study of bone healing.

#### 2.1.1. Inflammation

McKibbin [3] cites observations of bone growth from injury to the periosteum and callus formation around the stump of an amputee as evidence that an initial healing response that includes some periosteal bony callus formation is possible without ongoing mechanical stimulation [3]. This suggests that mechanical stimulation is not required during the inflammatory phase. Moreover, Hankmeier et al. [28] reported that mechanical stimulation delayed the invasion of macrophages during inflammation. After two weeks, Epari et al. [4] reported higher amounts of haematoma remnants in flexibly fixed ovine fractures compared to those more rigidly fixed. This suggests that the transition from inflammation to proliferation may even be hampered by mechanical stimulation.

#### 2.1.2. Proliferation

The proliferation phase is marked by the growth of callus tissue adjacent to the fracture. Tufekci et al. [17] demonstrated that mechanical stimulation applied only during the proliferation phase (days 5 to 21) significantly promoted the healing in comparison to unstimulated controls [17]. Recently, Glatt et al. [29] reported accelerated healing in animals treated with a fixator that was flexible for the first three weeks post-op and subsequently rigid for five weeks, in comparison to animals treated with a fixator that was rigid for the entire duration of the study. Furthermore, Epari et al. [4] showed on histological slices that, three weeks post-op, the callus formation was more pronounced for the fractures fixed with flexible fixation which allowed for higher amplitude of stimulation during the proliferation phase. Additionally, the callus formation on a far cortex—where, due to the bending of the fixation, the amplitude of stimulation was higher –was larger. These results suggest that mechanical stimulation is required during the proliferation phase and that higher amplitudes stimulations are beneficial.

#### 2.1.3. Consolidation

The consolidation phase is characterised by the narrowing of the gap between the two externally formed bony callus tissues. Hente et al. [15] showed that constant stimulation with the same amplitude generated a massive callus response but it failed to consolidate the fracture. The initial magnitude was kept at the same amplitude for the entire duration of the experiment; thereby the stimulation was constantly damaging the tissue in the fracture gap, preventing bridging [15]. Gardner et al. [30] showed that a progressive decrease in the amplitude of stimulation that was applied with an active fixator (from the fifth week onwards) resulted in a more rapid regaining of fracture stiffness than in the case of a constant level of stimulation. However, Tufekci et al. [17] showed that complete abolition of motion three weeks post-op led to higher torsional strength (nine weeks post-op), then a progressive decrease in the amplitude from the fourth week onwards [17]. This suggests that mechanical stimulation is not required at all during the consolidation phase.

#### 2.1.4. Remodelling

Most studies do not address or provide little insight into the optimum mechanical conditions during the remodelling phase. This is because full function is achieved after bony bridging of the callus which proceeds resorption and remodelling of the callus from woven bone into mature bone. However, the studies of Claes et al. [10] and Willie et al. [31] demonstrated the benefit of late dynamization. This suggests that removal of the fixation may enhance the remodelling process.

### 2.2. How Should Stimulation Be Applied?

In the previous section, we surmised the optimum mechanical environment during the different temporal phases of bone healing based on recent experimental investigations. In this section, we address how the stimulation should be applied in terms of the distribution of stimulatory cycles and rest periods.

In natural healing, the stimulation of interfragmentary movements occurs with the functional loading and movement of the individual. Pre-clinical studies with active fixation and specially configured bone defect models afford the ability to control the distribution of stimuli and investigate the effect of changes in the number of cycles and length of rest periods.

Using active fixation in an ovine bone defect model, Hente and Perren [16] demonstrated that continuous stimulation—with only an 8.6 s pause between stimulation cycles—prevents callus formation, while a pause of 1.4 min or even 2.4 h results in callus formation. The group with the 2.4 h pause received only ten stimulation cycles per day, yet that group developed the highest value of in vivo fracture stiffness [16]. This demonstrates that rest periods are essential for tissue formation.

Hente and Perren [16] used a defect model that consisted of a bone fragment in the shape of a wedge that was cut out from a tibial diaphysis and was connected to an external actuator that tilted the wedge around its apex. This configuration minimized the effect of functional loading on the experimental defect. Generally, in fracture healing models (complete osteotomy), capturing the influence of the stimulation/rest ratio is technically challenging during experiments due to the animal’s uncontrolled functional loading (weight bearing). This undesired mechanical stimulation may blur the effects of superimposed loading protocols being investigated with an active fixation.

Tufekci et al. [17] circumvented this problem by instrumenting an active motorized fixator on a double osteotomy model, thus eliminating the effect of functional loading. Barcik et al. [32] integrated a force sensor into the Tufekci fixator in order to continuously measure the stiffness of the newly formed tissue, and subsequently applied this system in a sheep model. These animals received a daily stimulation protocol that consisted of 1000 loading cycles that were evenly distributed over 12 h and were followed by 12 h of rest to resemble a simplified clinical situation with an active phase during the day (12 h) and an overnight rest (12 h). It was observed that the fracture stiffness decreased during the daily onset of stimulation. Bishop [33] and Meyers et al. [34] also observed a decay in fracture stiffness at the beginning of a daily stimulation batch. Meyers et al. attributed these changes to interstitial fluid flow [34]. However, this effect may also reflect the hypothesis of Perren where mechanical stimulation disrupts newly formed callus tissue [35].

It is unknown how long the effect of a single stimulation batch lasts before the next stimulation cycles should be applied; however, based on recent results [16,32,33] we can assume that a prolonged resting period (in the order of days rather than hours) would be beneficial. However, the exact ratio of stimulation and resting requires further animal experiments.

## 3. Hypothesis

Based on the result of studies presented in the prior sections, we hypothesise that the optimal delivery of mechanical stimulation follows two principles:

- The mechanical stimulation should be applied predominantly during the proliferative phase to boost callus formation and should be stopped soon after the deflection of the healing curve to allow for consolidation.
- When stimulation is applied, sufficient resting time should be provided between the stimulatory events to allow for callus formation.

**Figure 2:**
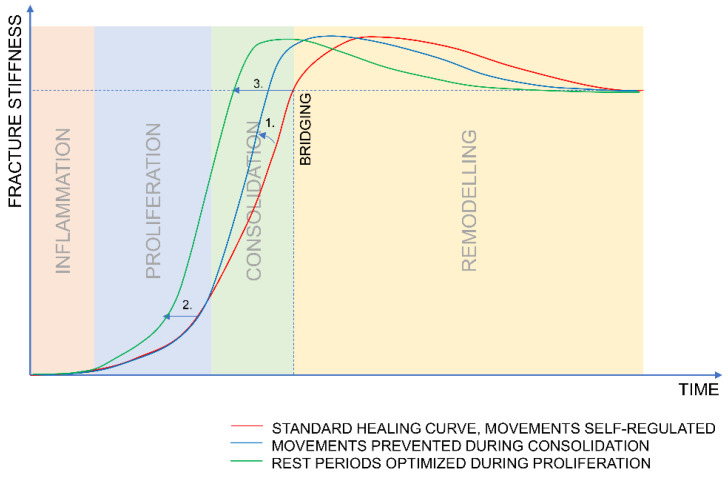
1. Halting the motion during the consolidation phase increases the rate of stiffness increase. 2. Increasing the rest periods during proliferation increases the rate of tissue formation and shortens the time to consolidation; 1. and 2. combined to shorten the overall time for bridging (3.).

Figure 2 illustrates the hypothesized benefits of the optimized stimulation protocol.

## 4. Discussion

In the previous sections we summarized the studies that guided us to the hypothesis that we formulated. In this section, we discuss the studies that may contradict it.

### 4.1. What Evidence Contradicts the Hypothesis?

We hypothesized that a high amplitude of stimulation applied in the early stage of healing is beneficial. However, Müller et al. [36] did not observe a difference in callus ex vivo stiffness between the animals that received a fixation that was constantly stiff during the entire study and the animals that received initially flexible fixation and subsequently stiffened three weeks post-op. Moreover, no difference was observed between the treated and the intact bones for both groups. This suggests that, at the endpoint of the experiment, both groups healed to the level that would allow for unrestricted physiological function. To determine if certain strategies accelerate or delay healing, we need to apply a reliable method for the continuous measurement of fracture stiffness to determine the healing rate over the experiment and the healing stage at the moment of euthanasia. Blind comparison of the results from post-mortem biomechanical tests may cause a significant misinterpretation, as the curves of fracture stiffness for different healing strategies intersect after bridging (Figure 2).

Moreover, Bartnikowski et al. [12] tested in a rat model the influence of stiffening the initially flexible implant at different timepoints and did not prove the benefit of this strategy in comparison to a constantly stiff fixator; however, stiffening the fixation did significantly improve healing compared to the more flexibly fixed control. In a clinical situation, we would expect more difference because of the lack of early stimulation.

Furthermore, we postulated that significant improvements may be achieved with different temporal distributions of stimulation during the proliferation phase. In contrast, Gardner et al. [37] did not observe significant differences in callus strength and stiffness when testing different ratios of stimulation and resting in mice. However, in this experiment, the animals’ activity was not monitored; therefore, the uncontrolled weight bearing might have influenced the mechanical environment in the fracture and blurred the difference between the study groups. This demonstrates the advantage of active fixation devices that can apply precisely defined amounts of stimulation not disturbed by the animal’s weight bearing.

### 4.2. How Does Clinical Treatment Compare?

The current clinical treatment of fractures best mirrors the passive “set and forget” approach to fracture fixation. Dynamization of nails is a common practice; however, rather than a mechanical dynamization that changes fixation stiffness, the benefit is typically derived from reducing the size of the fracture gap [10,11].

**Figure 3:**
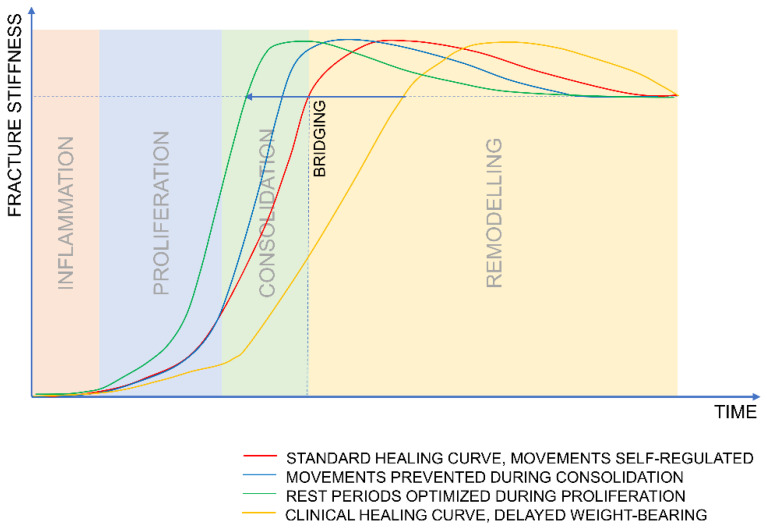
Hypothesized benefit of the optimized stimulation protocol in reducing healing time in comparison to current clinical practice.

Due to concerns over implant failure, surgeons are typically conservative with respect to prescription of post-operative weight bearing. Combined with often too-stiff fixation devices (locking plates), a common outcome is insufficient callus formation [38,39]. Post-operative weight bearing is encouraged only once callus formation can be visualized (consolidation phase) which may be the time when fracture motion should be limited to support bridging. This, combined with the initial lack of stimulation, may limit the formation of fracture callus and therefore delay the whole healing process (Figure 3).

### 4.3. Design of an Experiment

**Figure 4:**
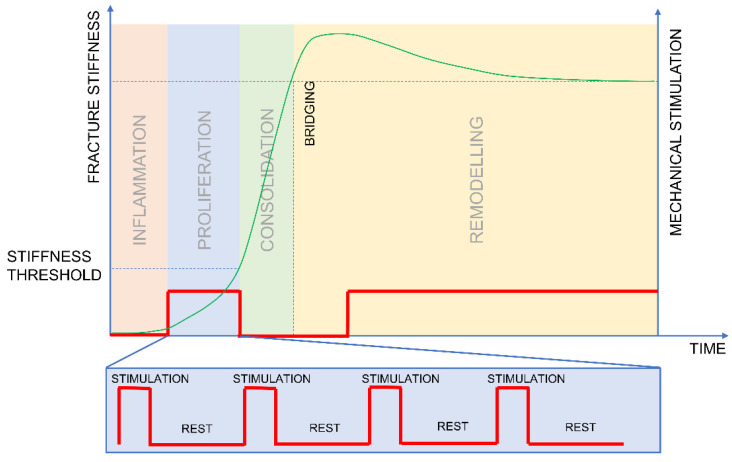
The protocol designed to test the hypothesis of the optimum delivery of mechanical stimulation.

Further experimental work needs to be conducted to verify the hypothesised optimal mechanical stimulation of fractures and the predicted benefits. Figure 4 depicts a protocol designed to test the hypothesis of the optimum delivery of mechanical stimulation. Stimulation should be applied in a sequence of a short loading and a prolonged rest (Figure 4) during the proliferation phase. The stimulation should be stopped when the value of fracture stiffness reaches the predefined threshold to allow for subsequent consolidation. The stimulation should be further allowed in the late stage of healing, as was documented by Claes et al. [10].

The experiment should be conducted on a large animal model because higher-order mammals (sheep, goats) more effectively mimic humans than rodents in terms of biomechanics [40]. The mechanical stimulation of the fracture should be realized with an active fixator. In contrast to passive fixators, where the amount of stimulation is only determined by an animal’s weight bearing, active ones allow the application of equal amounts of stimulation for all animals within a study group. The proposed protocol consists of defined amounts of stimulation and resting. Therefore, it is crucial to use a fracture model that precisely executes the stimulation protocol and excludes the influence of physiological loading on the experimental defect; this ensures that the fracture repair tissue is not disturbed during resting [16,17].

Our group recently developed a system [41] for the continuous measurement of repair tissue stiffness. Using this model, we can assess the mechanical competence of fracture repair tissue without sacrificing multiple animals at different time points. This system is a modification of the Tufekci model [17] and therefore eliminates the influence of physiological loading on the experimental defect.

In the proposed stimulation protocol, the mechanical stimulation is only applied during the proliferation phase until the healing curve reaches a certain stiffness threshold and deflects (Figure 4). Based on the results reported by Goodship et al. [42] and Bishop [33] we can estimate that the deflection point is between 500 and 1000 N/mm of fracture stiffness.

To test the proposed hypothesis in vivo, we suggest the following study groups:

- Group 1—mimics clinical scenario—no stimulation until callus formation is visualized on an X-ray. The stimulation should be applied as batches of 100 cycles applied every hour, yielding 1200 cycles over 12 h. This protocol should mimic a simplified clinical scenario; therefore, no stimulation is applied overnight.
- Group 2—inverse/reverse dynamization group—stimulation applied from the fifth postoperative day until the stiffness reaches the threshold of 750 N/mm. The stimulation should be applied as batches of 100 cycles applied every hour, yielding 1200 cycles over 12 h; no stimulation is applied overnight.
- Group 3—prolonged resting periods—stimulation applied from the fifth postoperative day until the stiffness reaches the threshold of 750 N/mm. The stimulation should be applied once a day in a batch of 1200 cycles.

In this configuration, Groups 2 and 3 will receive the same number of cycles per day but the resting time will be different.

The model described by Barcik et al. [32] enables the measurement of fracture stiffness. The device measures the force in response to the applied displacement. Therefore, it is impossible to measure stiffness without stimulating the fracture. Consequently, the continuous monitoring of the mechanical competence of fracture repair tissue is not possible during the periods when no stimulation is applied.

We suggest that this limitation could be overcome by predicting callus strength using computed tomography (CT) [43]. However, a direct application of this method is not trivial in the case of the proposed experiment. The metal hardware of an active fixator may introduce artefacts into the CT image, which may prevent the reliable estimation of the mechanical competence of fracture callus. Further work is needed to address these limitations and ensure that they do not obscure the experimental findings.

In parallel with animal studies, the impact of local mechanical conditions on healing progression has been investigated using in silico models [44,45,46]. Multiple computational studies have focused on the impact of clinical parameters on healing progression, such as the fracture gap size [47,48], type and configuration of the fixation [49] or the fixator’s stiffness [50,51]. However, the aspects of the temporal distribution of mechanical stimulation have not been frequently studied in silico. Recently, Fu et al. [52] investigated the impact of dynamization during different stages of healing; they demonstrated the potential for influencing healing using different timings for dynamization. Further computational simulations that are validated with in vivo models may help to design and test stimulation protocols that have the potential to decrease fracture-healing time.

## 5. Conclusions

Reflecting on the last 70 years of research in the field of bone repair, we pose a provocative question of whether healing time can be significantly reduced by the modulation of the mechanical environment in the fracture gap. Decades of in vivo research led to the development of the biological internal fixation [53] that significantly improved fracture biology. However, can we go further and not only use mechanics to prevent the delay of healing but use it in order to accelerate healing?

Based on the reviewed literature, we hypothesized that the manipulation of the local mechanical environment has the potential to significantly decrease the healing time for fracture patients. Further experiments should be conducted to determine the best parameters for stopping the interfragmentary motion to allow for consolidation and to establish the best stimulation/rest ratio. Even though the optimal delivery of mechanical stimulation presented here may appear to be clinically unfeasible with existing hardware, this may soon change with recent technological advancements and the development of active implants [8,36,54]. When the technological obstacles have been overcome, we need to be ready with the understanding to maximise their potential.

## Data Availability

Not applicable.
